# Team Social Media Usage and Team Creativity: The Role of Team Knowledge Sharing and Team-Member Exchange

**DOI:** 10.3389/fpsyg.2021.755208

**Published:** 2021-12-06

**Authors:** Hui Wang, Yuting Xiao, Xinwen Su, Xiangqing Li

**Affiliations:** Business School, Xiangtan University, Xiangtan, China

**Keywords:** work-related team social media usage, relationship-related team social media usage, team knowledge sharing, team-member exchange, team creativity

## Abstract

Given that work teams have been widely used in a variety of organizations to complete critical tasks and that the use of social media in work teams has been growing, investigating whether and how team social media usage (TSMU) affects team creativity is imperative. However, little research has empirically explored how TSMU affects team creativity. This study divides TSMU into two categories, namely, work-related TSMU and relationship-related TSMU. Basing on communication visibility theory and social exchange theory, this study constructs a moderating mediation model to understand how TSMU affects team creativity. In this model, team knowledge sharing is used as mediating role and team-member exchange (TMX) is used as moderating role. Two-wave research data collected from 641 employees in 102 work teams in Chinese organizations are used for regression analysis. Results show that (1) Work-related TSMU and relationship-related TSMU are positively affect team creativity. (2) Team knowledge sharing plays a partly mediating effect on the relationship between work-related TSMU and team creativity and that between relationship-related TSMU and team creativity. (3) TMX not only positively moderates the indirect effect of work-related TSMU and relationship-related TSMU on team creativity through team knowledge sharing. Theoretical and practical implications are also discussed.

## Introduction

Creativity—the joint novelty and usefulness of ideas regarding products, services, and processes (Zhou and Hoever, 2014; Amabile and Pratt, 2016)—is critical for organizations to survive and attain a competitive advantage in today’s rapidly changing environment (Shin et al., 2012). With the increasing complexity of organizational tasks, work teams have been widely used in a variety of organizations to complete critical tasks (Gino et al., 2010; Mathieu et al., 2017) and stay competitive (Pirola-Merlo and Mann, 2004; Zhou and Hoever, 2014). Following the conception of creativity, team creativity is defined as the joint novelty and usefulness of a final idea developed by a group of people (Hoever et al., 2012). Thus, how to improve team creativity is not only the main goal pursued by enterprises but also an important research topic.

In the review of prior literature, many scholars have investigated the antecedents of team creativity. In particular, the antecedents of team creativity mainly include individual characteristics, team factors, organizational factors, task characteristics, and leader factors (Hoever et al., 2012; Zhou and Hoever, 2014; Lee et al., 2016; Van Knippenberg, 2017; Wang et al., 2019; Liu et al., 2020). Although most prior studies have made outstanding contributions to the team creativity literature, one of the important aspects ignored by scholars is whether team social media usage (TSMU) affects team creativity or not. Social media, such as QQ, Wechat, DingTalk, and Weibo in China, has been widely used for employees to communicate within the work team. Unlike traditional face-to-face communication, social media has the character of affordance, which enables employees to communicate with others everywhere and anytime (Rice et al., 2017; Sun et al., 2019). For example, Wechat group—one of the social media popular in China—enables team members to post, edit, and sort text and files about work task linked to themselves or others in the Wechat group, and others can immediately view the messages, text, and files at the same time. In addition, when team members are in different places, Tencent meeting—another social media popular in China—enables teams to implement online meetings to discuss work items. Some researchers held the perception that social media usage can help individuals generate novel and useful ideas concerning products, services, and work methods (Leonardi, 2014; Cheng and Krumwiede, 2018; Wushe and Shenje, 2019; Chen et al., 2020; Korzynski et al., 2020). However, research on the relationship of TSMU and team creativity has been scarce. Team creativity is not simply the aggregation of ideas generated by individual members; rather, it involves team members collectively processing information, considering disparate views, and eventually producing creative outcomes. Therefore, the effect of TSMU on team creativity is a more complex process.

To address this theoretical gap, this study develop and test a theoretical model to test how and when TSMU benefits team creativity. This study divides TSMU into two categories, namely, work-related TSMU and relationship-related TSMU. The former normally emphasizes completing work tasks efficiently, whereas the latter focuses on developing personal relationships (Kwon and Wen, 2010; Sun and Shang, 2014; Van Zoonen et al., 2017; Liu et al., 2020; Ma et al., 2020). Communication visibility theory indicates that social media make communications between coworkers even more visible to those not directly involved than preceding communication technologies (Leonardi and Treem, 2012), that is to say, once the content and networks of social media users become visible to third parties, the third-party observers can improve their knowledge of “who knows what” and “who knows whom” (Leonardi, 2014, 2017). Basing on communication visibility theory, this study make effort to explore the mediating effect of team knowledge sharing on the relationship between TSMU and team creativity. On one hand, work-related TSMU means that team members discuss work-related information (e.g., work plan, work process) on social media. The visibility of work-related information enables employees to accurately understand the work progress of different teammates and their fields of expertise. Based on this, employees can accurately share knowledge with different teammates, so as to promote the efficient completion of team tasks. On other hand, relationship-related TSMU means that team members use social media to post more personal information, such as personal interest and preference. The visibility of personal interest and preference promote employees to share knowledge that teammates interested, so as to maintain and enhance the relationship with them. Furthermore, team knowledge sharing results in integration of existing knowledge and ideas within teams for enhancing team creativity (Men et al., 2017).

As discussed above, social media usage provides technical support for team knowledge sharing, which makes team knowledge sharing more convenient. However, team members’ knowledge sharing willingness is also vital to team knowledge sharing. According to social exchange theory, TMX, which defined as “the individual member’s perception of his or her exchange relationship with the peer group as a whole” (Seers, 1989, p. 119), reflect the quality of working relationships within a team. Low levels of TMX reflect troubled relationships with other team members, thus individuals willingness to share knowledge within team is also low. While higher levels of TMX reflect more congenial and more reliable coworker relationships, which leads to higher willingness to share knowledge (De Vries et al., 2006; Baek et al., 2018). Consequently, this study take step to explore the moderating role of TMX on the relationship between TSMU and team knowledge sharing, and further investigates the moderating role of TMX on the indirect effect of TSMU on team creativity through team knowledge sharing.

**FIGURE 1:**
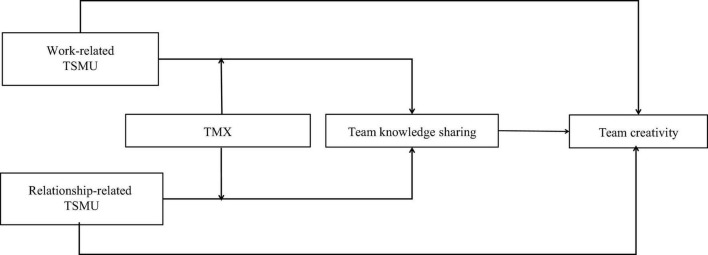
Theoretical model.

The theoretical framework is presented in Figure 1. This study has the following contributions. First, this study contributes to the team creativity literature by revealing the relationship between TSMU and team creativity. Although some previous research had examined the relationship of social media usage and individual creativity (Leonardi, 2014; Ding et al., 2019; Chen et al., 2020; Korzynski et al., 2020), but few literature focused on the effect of TSMU on team creativity. Second, this study contributes to communication visibility theory by revealing the mediating role of team knowledge sharing between the relationship of TSMU and team creativity. Leonardi (2014) had proposed communication visibility theory, and the communication visibility theory provides a new perspective for the research fields of organizational behavior and knowledge management. However, few researchers have conducted empirical tests on it (Engelbrecht et al., 2019). Third, this study explores the moderating role of TMX on the indirect effect of TSMU on team creativity via team knowledge sharing based on social exchange theory. Thus, this study also broadens research on the boundary condition of the indirect effect of TSMU on team creativity via team knowledge sharing.

## Theoretical Background and Hypotheses

### Team Social Media Usage

Kaplan and Haenlein (2010) defined social media as a group of internet-based applications that allow users to create content, share knowledge, and transmit information (Kaplan and Haenlein, 2010). Yates and Paquette (2011) stated that social media is an emerging technology with the potential to allow for flexibility, adaptability, and boundary spanning functionality demanded by response organizations for their information systems. Organizations are increasingly using social media as a part of their organizational communication strategy (Leonardi et al., 2013; Cardon and Marshall, 2014; Brooks, 2015; Bhimani et al., 2019; Sun et al., 2019; Venkitachalam and Bosua, 2019). Social media used in organizations include two types. One type is public social media platform, such as Facebook, Youtube, Twitter, Instagram, QQ, Wechat, and Weibo. Another type is internal social media platform, such as DingTalk, DongTalk, and Think Tomorrow. Specifically, enterprise social media is defined as “web-based platforms that allow workers to (1) communicate messages with specific colleagues or send mass messages to everyone in the organization; (2) post, edit, and sort text or files linked to themselves or others; and (3) browse the messages, connections, text, and files communicated, posted, edited, and sorted by others in the organization at any time of their choosing (Leonardi et al., 2013). Furthermore, enterprise social media usage has been divided into work-oriented enterprise social media usage and relationship-oriented enterprise social media usage (Kwon and Wen, 2010; Sun and Shang, 2014; Van Zoonen et al., 2017; Liu et al., 2020; Ma et al., 2020). The former normally emphasizes completing work tasks efficiently, whereas the latter focuses on building and maintaining relationships.

Given that work teams have been widely used in enterprises to complete critical tasks, and basing on previous literature on enterprise social media usage (Kwon and Wen, 2010; Liu et al., 2014, 2020; Sun and Shang, 2014; Van Zoonen et al., 2017; Ma et al., 2020), this study defines TSMU as the extent to which team members usage of TSMU either for tasks or for relationships. According to this concept, TSMU is divided into two types: work-related TSMU and relationship-related TSMU. The difference between the two is reflected in three aspects: motivation, behavior, and social ties. First, the motivation of work-related TSMU focuses on completing team work efficiently, whereas that of relationship-related TSMU emphasizes building and maintaining team members’ relationships. Second, work-related TSMU includes behaviors such as (1) sharing work-plans and task-objectives information, (2) discussing work item information, and (3) discussing work process information with teammates. Relationship-related TSMU includes behaviors such as (1) concern for teammates, (2) encouraging teammates, and (3) supporting one another within the team. Third, team members can build instrumental ties with one another through work-related TSMU, whereas they can build expressive ties through relationship-related TSMU. Although work-related TSMU and relationship-related TSMU differ in motivation, behavior, and social ties, they are essentially similar in substance, that is, they aim to connect people and disseminate and share information (Ng et al., 2016).

### Team Social Media Usage and Team Creativity

Team creativity is defined as the development of novel and useful ideas which are relevant to products, services, processes, and procedures by a team of employees working together (Shin and Zhou, 2007; Farh et al., 2010; Men et al., 2017). Team creativity is not simply the aggregation of ideas generated by individual members; what’s more, it refers to team members considering disparate views, collectively processing information, and eventually producing creative outcomes (Dong et al., 2016).

Communication visibility theory argues that transparent communication within a team enables employees to improve their awareness of “who knows what” and “who knows whom” (Leonardi, 2014). According to communication visibility theory, TSMU could facilitate team creativity by message transparency and network translucence (Leonardi, 2014). Work-related TSMU could increase message transparency which facilitates team members generate ideas, coordinate with each other, and complete tasks, whereas relationship-related TSMU could enhance network translucence which allows team members to demonstrate the social ties with each other, thereby building and maintaining network relationships.

First, work-related TSMU means that team members use social media to communicate work-related information, such as work content, work process and work procedure. Social media can make previously created and published content visible and permanently accessible, which ensures message transparency in the team (Wagner and Majchrzak, 2007). For this reason, teams with high work-related TSMU could afford more visible work-related information which is useful for team members to complete tasks, and employees are easier to seek useful knowledge and experience from these information (Leonardi and Meyer, 2015; Leonardi and Vaast, 2017). In other words, work-related TSMU enables employees obtain enough work-related information to decide how to accomplish tasks creatively. What’s more, team members’ discussion on work and the collision of different ideas are conducive to stimulate team innovation.

Second, relationship-related TSMU means that team members use social media to build and maintain relationship with each other, such as concerning teammates, encouraging teammates and supporting teammates. Social media has the characteristics of network transparency, that is to say, the behaviors, preferences, and communication networks of team members are visualized by social media (Leonardi, 2014). Thus, employees can keep abreast of their teammates’ personal information (such as interests, preferences, mood, etc.) (Ellison et al., 2011), so as to provide emotional support for teammates (Schreurs et al., 2014; Wang et al., 2019). For example, individual brows his colleagues personal homepage (such as Facebook) or microblog (such as Twitter and Sina Weibo) to learn about their personal information. Furthermore, network translucency enables employees to expand the range of members through reactivate dormant ties and maintain a set of “latent ties” (Ellison et al., 2011; Levin et al., 2011; Ou and Davison, 2016; Cheng and Krumwiede, 2018; Pitafi et al., 2018; Wushe and Shenje, 2019). Therefore, relationship-related TSMU will lead to closer network connection in the team, so as to ensure close connection among team members and enhance team creativity (Joo et al., 2012).

In summary, from the perspective of communication visibility theory, work-related TSMU contributes to team creativity as message transparency helps team members understand their task better and collaborate better, while relationship-related TSMU contributes to team creativity because of network translucence in the team enhances the team cohesion.

***H1a:*** *Work-related TSMU is positively related to team creativity.*

***H1b:*** *Relationship-related TSMU is positively related to team creativity.*

### Team Social Media Usage and Team Knowledge Sharing

Knowledge sharing is a process in which an individual shares their relevant knowledge, ideas, suggestions, and skills with others (Srivastava et al., 2006). Knowledge sharing occurs through communication and information exchange between individuals. Ipe (2003) argued, knowledge sharing in team is a complex process affected by organizational, individual, technical, and cultural factors. TSMU refers to social media that are used to communicate with one another in work team for completing work task and enhancing team members relationship. Based on communication visibility theory, we predict that both work-related TSMU and relationship-related TSMU are positively related to team knowledge sharing.

Work-related TSMU is assumed to constitute an effective predictor of team knowledge sharing. Work-related TSMU means team members use social media to (1) share work-plans and task-objectives information, (2) discuss work item information, and (3) discuss work process information with teammates. According to communication visibility theory, work-related TSMU helps employees quickly access information “who knows what,” which is benefit for knowledge sharing (Leonardi, 2014; Nduhura and Michael, 2017). Specifically, the visibility of work-related information enables employees to accurately understand the work progress of different teammates and their fields of expertise, then, they can accurately share knowledge with different teammates to complete team tasks efficiently. Second, TSMU focuses on conducting communication and exchanges related to work tasks. As team members communicate more frequently about their work or ideas, the team members’ trust in each other will be deepen (Butler and Cantrell, 1994). Trust is considered to be a key factor to reduce complexity, risk and uncertainty, so as to form an atmosphere of positive cooperation among team members (Jarvenpaa et al., 1998; Baba, 1999; Mohammad et al., 2017). Thus, when team members trust each other, they are willing to share knowledge with each other (Chow and Chan, 2008; Zhou and Hoever, 2014). Third, due to the visibility of information, the chat records of employees on social media (QQ, Wechat, Ding talk) can be seen by the leaders. In order to improve the impression in the minds of the leaders, employees are more likely to make knowledge sharing, which is beneficial to the team.

***H2a:*** *Work-related TSMU is positively related to team knowledge sharing.*

Relationship-related TSMU is assumed to positively relate to team knowledge sharing. First, relationship-related TSMU mainly refers to that team members tend to use social media to post personal related information (such as personal interests and life status) and prefer to pay attention to the status of other members (Liu et al., 2014; Huang and Liu, 2017). For example, individuals either post some information and links they pay attention to on their Wechat or write blog and post photo about their life on their Weibo. Team members use social media for emotional communication and provide emotional support and encouragement to each other, which promotes mutual understanding, maintenance and development of the relationship between team members. Therefore, employees can timely understand the problems encountered by other team members and share valuable knowledge and information (Chia et al., 2006). Second, team members use social media to communicate, provide encouragement and support to each other, so as to enhance mutual trust, create a working environment of common trust (Mooradian et al., 2006; Lombardi et al., 2019). In this atmosphere, employees are willing and able to share knowledge that is really beneficial to other members.

***H2b:*** *Relationship-related TSMU is positively related to team knowledge sharing.*

### Team Knowledge Sharing and Team Creativity

Team creativity, which refers to the joint novelty and usefulness of ideas regarding products, processes, and services (Zhou and Hoever, 2014; Amabile and Pratt, 2016), is a result of the interactions of team members (Bodla et al., 2016; Dong et al., 2016). According to the componential theory of creativity, creativity includes three important components: expertise, creative-thinking skill, and intrinsic task motivation (Amabile, 1996).

Knowledge sharing is referred to as the provision or exchange of ideas and information (Cummings, 2004; Gagné et al., 2019). This study infers that team knowledge sharing can positively predict team creativity for the following reasons. First, Knowledge is the basis of innovation. Team knowledge includes explicit knowledge and tacit knowledge, which further developed from the knowledge contribution of employees and their interaction in the workplace (Venkitachalam and Busch, 2012). Team knowledge sharing can improve the repository of available explicit knowledge and tacit knowledge in the team (Gardner et al., 2012), and expand the knowledge stock of the team. Furthermore, the team knowledge stock can provide more opportunities to reorganize existing information and ideas (Sung and Choi, 2012; Huang et al., 2014). Therefore, the team can use and integrate resources to creatively complete tasks, such as developing new products or procedures. Second, knowledge sharing can increase the mutual understanding of team members and facilitate the motivation to gain insights from other team members to broaden their scope of knowledge (Gong et al., 2013), which are important sources of team creativity. With the abundant knowledge through sharing with others, team members are more likely to utilize a variety of perspectives, ideas, and expertise of other team members to generate novel and creative ideas in a context requiring creativity (Shin et al., 2012; Zhang et al., 2019).

***H3:*** *Team knowledge sharing is positively related to team creativity.*

### Mediating Role of Team Knowledge Sharing Between Team Social Media Usage and Team Creativity

Given that both work-related TSMU and relationship-related TSMU are positively related to team knowledge sharing, and team knowledge sharing is vita for team creativity. We propose that both work-related TSMU and relationship-related TSMU indirect affect team creativity via team knowledge sharing.

First, according to communication visibility theory, work-related TSMU can positively predict team creativity via promote team knowledge sharing. As Leonardi (2014) argued that message transparency enabled by social media can allow employees and third party to literally see the content of the exchanged messages among their coworkers. Work-related TSMU affords the possibility of making visible the communicative activities in which one engages at work, such as work content, work method, work progress, and work results. Thus, the team knowledge distribution structure and the location of knowledge sources (“who knows what.”) become clear to team members (Bezrukova et al., 2012; Wagner et al., 2014; Ratasuk and Charoensukmongkol, 2020), so that team members can accurately recognize their advantages of professional knowledge and information resource, and then share knowledge and information in the team. In this way, the knowledge stock of the team is increasing, and sufficient knowledge resources help to improve the team creativity (Shin et al., 2012; Gong et al., 2013; Huang et al., 2014; Hu and Randel, 2014; Bodla et al., 2016). In addition, when an individual in the team encounters difficulties at work, he can timely seek help from his colleagues through social media, so that other members can understand his problems in time, and help him solve problems by sharing knowledge, experience and methods, so as to promote the overall work of the team and enhance the creativity of the team. Thus, work-related TSMU indirect affects team creativity via team knowledge sharing.

***H4a:*** *Team knowledge sharing mediates the relationship between work-related TSMU and team creativity.*

Second, relationship-oriented TSMU emphasizes building and maintaining team members’ relationships. Relationship-related TSMU ensures network translucence by visualizing the behaviors, preferences, and communication networks of team members (Leonardi, 2014), so as to facilitate them to build, maintain and enhance their social relationship (Ellison et al., 2011). Specifically, team members use social media to post personal and general information, such as personal interests, preference and life status. Therefore, employees can be aware of colleagues’ interests and hobbies, build and maintain relationships with colleagues by sharing information related to colleagues’ interests and hobbies (Chia et al., 2006; Liu et al., 2014). As a result, team members’ trust in each other will be deepen (Butler and Cantrell, 1994; Robertson and Kee, 2017; Yoo et al., 2019), which enhance team members’ knowledge sharing. In turn, increased knowledge stock enables the team to utilize and integrate the resources to accomplish complex tasks creatively, such as developing new products or promoting procedures (Gardner et al., 2012; Dong et al., 2016; Men et al., 2018). Thus, relationship-related TSMU indirect affects team creativity via team knowledge sharing.

***H4b:*** *Team knowledge sharing mediates the relationship between relationship-related TSMU and team creativity.*

### Moderating Role of Team-Member Exchange

Based on social exchange theory, Seers (1989) proposed the concept of TMX, which is the process of reciprocal exchange between team members, including a member offering help, ideas, and feedback for others and the degree of obtaining information, help, and recognition from other members. TMX reflects an individual’s overall perception of the quality of the work relationship within the team. The growth of TMX is based on interaction among members, and it affects team members’ attitude and behaviors (Seers et al., 1995). Low levels of TMX may reflect troubled relationships with other team members, and high levels of TMX may reflect more congenial and more reliable coworker relationships. Previous studies have indicated that high-quality TMX generally results in high levels of exchange in resources, support, and assistance for the completion of tasks among team members (Scott and Bruce, 1994; Liden et al., 2000; Barron and Chou, 2016; Wu et al., 2018; Rutishauser and Sender, 2019). In this study, we consider the potential moderating role of TMX between the relationship of TSMU and knowledge sharing.

Team members perceiving high-quality TMX tend to think that the relationship among team members is harmonious which can lead to knowledge sharing (Banks et al., 2013; Barron and Chou, 2016). In other words, employees perceiving high-quality TMX own more reliable colleague relationships, which helps to deeper trust and demonstrate close psychological connections (Schermuly and Meyer, 2016; Wu et al., 2018). Thus, in the situation of work-related TSMU, team members with high-quality TMX are more willing to share knowledge to promote the completion of team tasks. While team members with low-quality TMX are less willing to share knowledge.

Meanwhile, in the situation of relationship-related TSMU, team members with high-quality TMX are more willing to actively pay attention to colleagues through social media, be aware of their interests and hobbies, and share knowledge with them more actively. On the contrary, team members with low-quality TMX, even if relationship-related TSMU provides a convenient platform for communication between them and their colleagues, they are pay less attention to the personal information released by their colleagues on social media, may weaken the positive effect of relationship-related TSMU on knowledge sharing. Therefore, we hypothesize:

***H5a:*** *TMX moderates the relationship between work-related TSMU and team knowledge sharing. Compared with low-quality TMX, the positive relationship between work-related TSMU and team knowledge sharing is stronger under high-quality TMX.*

***H5b:*** *TMX moderates the relationship between relationship-related TSMU and team knowledge sharing. Compared with low-quality TMX, the positive relationship between relationship-related TSMU and team knowledge sharing is stronger under high-quality TMX.*

### Moderated Mediating Effect

Hypothesis 4a explains the mediating role of team knowledge sharing between work-related TSMU and team creativity, and Hypothesis 4b explains the mediating role of team knowledge sharing between relationship-related TSMU and team creativity. Hypothesis 5a illustrates the moderating effect of TMX on the relationship between work-related TSMU and team knowledge sharing, and Hypothesis 5b illustrates the moderating effect of TMX on the relationship between relationship-related TSMU and team knowledge sharing. Based on the above discussions, TMX positively moderates both the mediating path of “work-related TSMU – team knowledge sharing – team creativity” and the mediating path of “relationship-related TSMU – team knowledge sharing – team creativity.” Thus, the following hypotheses are proposed:

***H6a:*** *TMX positively moderates the indirect effect of work-related TSMU on team creativity through team knowledge sharing. That is, the higher the TMX, the greater the mediating effect of team knowledge sharing.*

***H6b:*** *TMX positively moderates the indirect effect of relationship-related TSMU on team creativity through team knowledge sharing. That is, the higher the TMX, the greater the mediating effect of team knowledge sharing.*

The theoretical model of this study is shown in Figure 1.

## Materials and Methods

### Sample and Procedure

This study examines the theoretical model using data collected from five large-scale technology enterprises in China. Participants are recruited as follows. First, five enterprises are identified through MBA alumni. Second, the human resource department directors of these two enterprises are contacted, and the purpose of data collection is explained. From these two enterprises, 113 work teams including 692 employees are recruited to participate in the questionnaire survey. A private email is then sent to all participants several days before the questionnaire survey to explain the research procedure and emphasize that the survey is for academic research purposes only and strictly under complete confidentiality.

In this study, two sets of questionnaires (individual- and team-level questionnaires) are designed. The questionnaire survey is composed of two stages: at Time 1, team members are required to complete individual-level questionnaire regarding predictor variables (task-oriented TSMU, relationship-oriented TSMU), mediating variable (team knowledge sharing), moderating variable (TMX), and individual-level control variables (age, gender, education). After a month, at Time 2, team leaders are required to complete team-level questionnaire regarding dependent variable (team creativity) and team-level control variables (team size, time since team was built).

A total of 692 individual-level questionnaires and 113 team-level questionnaires are finally collected. Among them, 51 individual-level questionnaires and 11 team-level questionnaires are discarded for missing data, leaving 641 valid individual-level questionnaires and 102 valid team-level questionnaires. Among the individual-level sample, 399 (62.25%) are males, and 244 (37.75%) are females. In terms of age, 91 (14.20%) are below 25 years old, 271 (42.28%) are between 25 and 30 years old, 154 (24.02%) are between 31 and 35 years old, 58 (9.05%) are between 36 and 40 years old, 45 (7.02%) are between 41 and 45 years old, and 22 (3.43%) are over 45 years old. In terms of education, 54 (0.08%) reach senior high school degree or below, 108 (16.85%) has a junior college degree, 347 (54.13%) has a bachelor’s degree, and 132 (20.60%) has a master’s degree or above. Among the team-level sample, in terms of team size, teams with 4–6 people account for 51.96% (53), teams with 7–9 people account for 37.25% (38), and teams with 10–12 people account for 10.78% (11). In terms of time since team was built, 23 teams have been in existence for 1–12 months, 34 teams have been in existence for 13–24 months, 16 teams have been in existence for 25–36 months, and 29 teams have been in existence for over 36 months.

### Measures

All scales’ items are originally developed in English and are therefore translated into Chinese. All scales’ items are measured on a 5-point Likert scale from 1 = “strongly disagree/unlikely” to 5 = “strongly agree/likely.”

### Team Social Media Usage

TSMU includes its two categories, work-related TSMU and relationship-related TSMU. The scale is adapted from Liu et al. (2014). The 10 items for measure work-related TSMU are as follows: members of my team mainly use social media to: (1) Make team work plans. (2) Discuss the way to carry out the work. (3) Set team work goals. (4) Promote team work process. (5) Deal with problems. (6) Identify potential problems. (7) Speed diagnose problem. (8) Discuss solutions to problems. (9) Make decision to choose problem solutions. (10) Integrate skills of all team members to solve problems. The Cronbach’s alpha for the scale of work-related TSMU is 0.979. The eight items for measuring relationship-related TSMU are as follows: members of my team mainly use social media to: (1) Provide emotional support for mutual assistance. (2) Motivate team members who feel frustrated. (3) Listen to members’ complaints. (4) Cultivate team cohesion. (5) Encourage team members to learn knowledge from one another. (6) Encourage team members to learn skills from one another. (7) Make friends within teams. (8) Set up social events with co-workers after working hours. The Cronbach’s alpha for the scale of relationship-related TSMU is 0.947.

### Team Knowledge Sharing

Team knowledge sharing is measured with an eight-item scale developed by Lu et al. (2006). The items such as: “I actively share my work-related knowledge with my colleagues in daily work,” “I can easily share with others rather than keep my work experience,” “I share useful work experience with my colleagues,” “As soon as i learn learn new knowledge useful to work, I promote it to others,” “So long as the other colleagues need it, I always tell whatever I know without any hoarding.” The Cronbach’s alpha for this scale is 0.943.

### Team-Member Exchange

Team-member exchange is measured with a 10-item scale developed by Seers (1989). Sample items are “Others let me know when I affect their work,” “I let others know when they affect my work.” The Cronbach’s alpha for this scale is 0.954.

### Team Creativity

Team creativity is measured with a four-item scale developed by Shin and Zhou (2007). The items are as follows: (1) My team always produce new ideas. (2) These new ideas are always useful. (3) These new ideas are crucial to my organization. (4) My team is creative. The Cronbach’s alpha for this scale is 0.914.

### Control Variables

Previous literature has shown that demographic variables and team characteristic variables may influence team creativity, including age, gender, education, team size and time since team was built (e.g., Shin and Zhou, 2007; Wang et al., 2016; Fong et al., 2018; Liu et al., 2020; Chen et al., 2021; Men and Jia, 2021). Thus, these variables are controlled in this study. Gender is measured as a dummy variable (1 = male, 2 = female). Age is divided into five levels (1 = under 25 years, 2 = 25–30 years, 3 = 31–35 years, 4 = 36–40 years, 5 = over 45 years). Education is divided into four levels (1 = senior high school or below, 2 = junior college, 3 = bachelor, 4 = postgraduate). Team size is divided into three levels (1 = 4–6 people, 2 = 7–9 people, 3 = 10–12 people). Time since team was built is divided into four levels (1 = 1–12 months, 2 = 13–24 months, 3 = 25–36 months, 4 = over 36 months).

### Data Analysis

SPSS 25.0 and Mplus 7.4 are used to analyze data. SPSS 25.0 is used to test the reliability of the five key variables and descriptive statistics and correlation analysis. Mplus 7.4 is used for confirmatory factor analysis (CFA), path analysis and aggregation analysis.

## Results

### Discriminant Validity

MPLUS7.4 is used to carry out CFA to test the discriminant validity of the variables. Compared to competition models, the theoretical five-factor model (work-related TSMU, relationship-related TSMU, team knowledge sharing, TMX, team creativity) has better fit to the data (χ^2^/df = 1.316, CFI = 0.943, TLI = 0.946, RMSEA = 0.056, SRMR = 0.051) (see Table 1). CFA results indicates that the theoretical five-factor model has satisfactory discriminant validity.

**TABLE 1:** Results of confirmatory factor analyses.

Model	Factors	χ^2^	df	χ^2^/df	RMSEA	TLI	CFI	SRMR
Five-factor model	W-TSMU, R-TSMU, TKS, TMX, TC	961.030	730	1.316	0.056	0.946	0.943	0.051
Four-factor model	W-TSMU + R-TSMU, TKS, TMX, TC	1341.776	734	1.828	0.090	0.859	0.850	0.079
	W-TSMU, R-TSMU, TKS + TMX, TC	1797.883	734	2.449	0.119	0.753	0.737	0.155
Three-factor model	W-TSMU + R-TSMU, TKS + TMX, TC	2177.615	737	2.955	0.138	0.665	0.645	0.166
Two-factor model	W-TSMU + R-TSMU + TKS +TMX, TC	2491.896	739	3.372	0.152	0.592	0.570	0.171
One-factor model	W-TSMU + R-TSMU + TKS + TMX + TC	2578.535	740	3.485	0.156	0.572	0.549	0.171
Unmeasured latent methods factor model		965.551	729	1,324	0.051	0.945	0.941	0.118

*W-TSMU, work-related TSMU; R-TSMU, relationship-related TSMU; TKS, team knowledge sharing; TC, team creativity.*

### Common Method Variance

First, anonymous measurement method, multi-source and two-wave design in survey are used to reduce CMV in the data collection. Second, the Harman single-factor test is used to assess the existence of CMV. The results show that the first factor solution in the exploratory factor analysis only explains 42.79% (<50%) loading, which proves the absence of CMV (Woszczynski and Whitman, 2004). Further, we conduct the unmeasured latent methods factor, that is, all items are loaded on both latent methods factor and trait factors (Podsakoff et al., 2003), to test CMV. A comparison of the latent methods factor model (χ^2^/*df* = 1.324, RMSEA = 0.051, CFI = 0.945, TLI = 0.941, SRMR = 0.118) and the theoretical five-factor model (χ^2^/df = 1.316, RMSEA = 0.056, CFI = 0.943, TLI = 0.946, SRMR = 0.051) indicates that the CFI becomes smaller (Cheung and Rensvold, 2002). Thus, CMV should not be a severe problem in this study.

### Data Aggregation

Work-related TSMU, relationship-related TSMU, team knowledge sharing, and TMX all are team-level variables, but the data of these variables are collected by individual-level questionnaires that are completed by team members. Thus, individual-level data should be aggregated at the team level. The results of data aggregation analyses are shown in Table 2. To examine the appropriateness of data aggregation, we first calculate the inter-rater agreement by calculating the rwg(j) values for each team (James et al., 1984). The mean rwg(j) of these four variables for the 102 teams are 0.878, 0.875, 0.900, and 0.758 (>0.70), achieving an acceptable level of inter-rater agreement. Second, we examine the intra-class correlation coefficients by calculating the ICC(1) and ICC(2) values of these four variables to show that the data are good for aggregation (James et al., 1984). The ICC(1) values of these four variables are 0.600, 0.295, 0.425, and 0.163 (>0.138). The ICC(2) values of these four variables are 0.906, 0.728, 0.834, and 0.554 (>0.50), indicating an acceptable reliability value for aggregated data (Kozlowski and Hattrup, 1992). Therefore, aggregating work-related TSMU, relationship-related TSMU, team knowledge sharing, and TMX as team-level variables is appropriate in this study.

**TABLE 2:** Results of data aggregation analyses.

Variable	Rwg	ICC (1)	ICC (2)
Work-related TSMU	0.878	0.600	0.906
Relationship-related TSMU	0.875	0.295	0.728
Team knowledge sharing	0.900	0.425	0.834
TMX	0.758	0.163	0.554

### Descriptive Statistics and Correlation Analysis

The results of descriptive statistics (mean, standard deviation) and correlation analysis (Pearson coefficient) are shown in Table 3. Work-related TSMU is positively correlated to team knowledge sharing (*r* = 0.677, *p* < 0.01) and team creativity (*r* = 0.742, *p* < 0.01). Relationship-related TSMU is positively correlated to team knowledge sharing (*r* = 0.596, *p* < 0.01) and team creativity (*r* = 0.703, *p* < 0.01). Team knowledge sharing is positively correlated to team creativity (*r* = 0.760, *p* < 0.01). The correlation between the key variables provides the initial support for the hypotheses. Given that the correlation between work-related TSMU and team creativity (*r* = 0.742, *p* < 0.01), the correlation between relationship-related TSMU and team creativity (*r* = 0.703, *p* < 0.01), and the correlation between team knowledge sharing and team creativity (*r* = 0.760, *p* < 0.01) are too high, there may be multi-collinearity problem. Thus, variance inflation factor (VIF) analysis was used to test multi-collinearity, and the results shows that VIF between work-related TSMU and team creativity is 2.283, VIF between relationship-related TSMU and team creativity is 1.918, and VIF between team knowledge sharing and team creativity is 1.990 (all meet the test standard of VIF < 10). The results indicates that there is no muti-collinearity between these variables in the theoretical model.

**TABLE 3:** Means, standard deviations (SD), and correlations.

Variable	Mean	*SD*	TTB	Team size	W-TSMU	R-TSMU	TKS	TMX	TC
TTB	2.559	1.157	1						
Team size	1.980	1.024	–0.166	1					
W-TSMU	3.501	0.734	0.089	0.143	1				
R-TSMU	3.623	0.466	0.004	0.305**	0.662**	1			
TKS	3.877	0.468	0.090	0.044	0.677**	0.596**	1		
TMX	3.387	0.499	–0.096	0.112	0.206*	0.198*	0.161	1	
TC	3.789	1.04	0.029	0.131	0.742**	0.703**	0.760**	0.090	1

N = 102 teams; *p < 0.05, **p < 0.01. TTB, time since team built; W-TSMU, work-related TSMU; R-TSMU, relationship-related TSMU; TKS, team knowledge sharing; TMX, team-member exchange TC, team creativity.

### Hypothesis Testing

**FIGURE 2:**
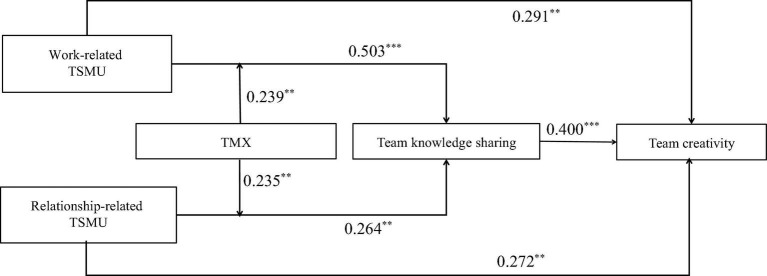
Results of theoretical model by using Mplus. *N* = 102 teams, ^**^p < 0.01, ^***^p < 0.001. Standardized path coefficients are reported.

First, we conduct the structural equation model (SEM) using Mplus to test the theoretical model. Figure 2 presents the results of SEM with the standardized coefficients. In Figure 2, both work-related TSMU and relationship-related TSMU are positively related to team creativity (β = 0.291, *p* < 0.01; β = 0.272, *p* < 0.01); thus, **H1a** and **H1b** are confirmed. Both work-related TSMU and relationship-related TSMU are positively related to team knowledge sharing (β = 0.503, *p* < 0.001; β = 0.264, *p* < 0.001); thus, **H2a** and **H2b** are confirmed. Team knowledge sharing is positively related to team creativity (β = 0.400, *p* < 0.001); thus, **H3** is confirmed.

Second, to test the mediating effects, we adopt bootstrap sampling method (bootstrap sample size = 5,000) recommended by MacKinnon et al. (2004) to generate the asymmetric confidence interval (CI) for indirect relationships. Table 4 presents the results of the bootstrap test. The indirect effect of “W-TSMU→TKS→TC” is significant (β = 0.201, CI is [0.026,0.340], excluding zero). The results suggest that team knowledge sharing plays a mediating role between work-related TSMU and team creativity. Thus, **H4a** is confirmed. The indirect effect of “R-TSMU→TKS→TC” is also significant (β = 0.106, CI is [0.001,0.210], excluding zero). The results suggest that team knowledge sharing plays a mediating role between relationship-related TSMU and team creativity. Thus, **H4b** is confirmed.

**TABLE 4:** Results of mediating effects test.

Effects	Estimate	*SE*	95% confidence interval	
			Lower limit	Upper limit
Total effect W-TSMU→TC	0.492	0.118	0.262	0.722
Direct effect W-TSMU→TC	0.291	0.132	0.031	0.550
Indirect effect W-TSMU→TKS→TC	0.201	0.071	0.062	0.340
Total effect R-TSMU→TC	0.378	0.087	0.207	0.549
Direct effect R-TSMU→TC	0.272	0.074	0.127	0.418
Indirect effect R-TSMU→TKS→TC	0.106	0.053	0.001	0.210

*W-TSMU, work-related TSMU; R-TSMU, relationship-related TSMU; TKS, team knowledge sharing; TC, team creativity.*

**FIGURE 3:**
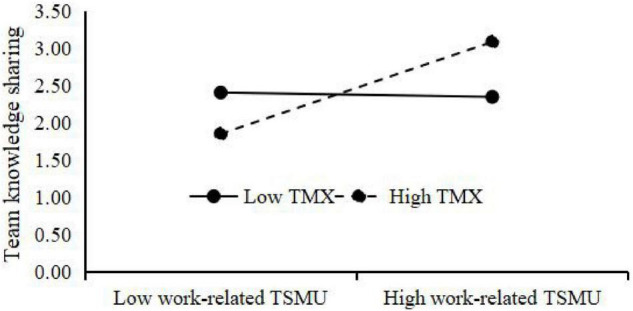
Interactive effect of work-related TSMU and TMX on team knowledge sharing.

**FIGURE 4:**
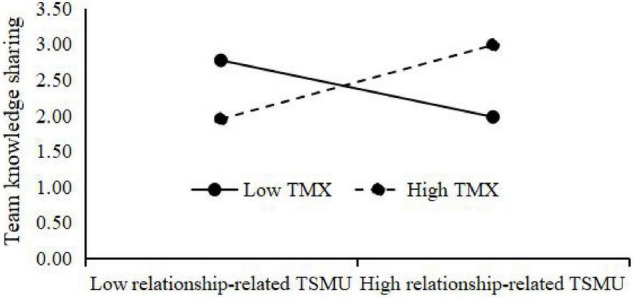
Interactive effect of relationship-related TSMU and TMX on team knowledge sharing.

Third, the results of the moderating effect test are shown in Figure 2. The interaction of relationship-related TSMU and TMX is significantly and positively related to team knowledge sharing (β = 0.239, *p* < 0.01), showing that TMX positively moderates the relationship of work-related TSMU and team knowledge sharing. The interaction of relationship-related TSMU and TMX is also significantly and positively related to team knowledge sharing (β = 0.235, *p* < 0.01), showing that TMX positively moderates the relationship of relationship-related TSMU and team knowledge sharing. In addition, to clearly show the moderating effect of TMX, this study adds and subtracts one standard deviation from the mean value of TMX and constructs two groups of high and low TMX. Then, the regressions are respectively calculated the regression equation, and the moderating effect diagram is drawn according to the regression coefficient (Figures 3, 4). Figure 3 shows that compared with low TMX, work-related TSMU has a greater effect on team knowledge sharing under high TMX, supporting **H5a**. Figure 4 shows that compared with low TMX, relationship-related TSMU has a greater effect on team knowledge sharing under high TMX, supporting **H5b**.

Fourth, the results of the moderated mediating effect test are shown in Table 5. At low TMX, the mediating effect of “W-TSMU→TKS→TC” is not significant (β = 0.086, CI is [–0.066,0.333], including zero). At high TMX, the mediating effect of “W-TSMU→TKS→TC” is significant (β = 0.179, CI is [0.335,1.035], excluding zero). The two groups show significant differences (β = 0.198, CI is [0.224,1.010], excluding zero). These results support that TMX positively moderates the mediating effect of “W-TSMU→TKS→TC”; thus confirming **H6a**. At low TMX, the mediating effect of “R-TSMU→TKS→TC” is not significant (β = –0.065, CI is [–0.425,0.279], including zero). At high TMX, the mediating effect of “R-TSMU→TKS→TC” is significant (β = 1.094, CI is [0.703,1.491], excluding zero). The two groups show significant differences (β = 1.159, CI is [0.602,1.748], excluding zero). These results support that TMX positively moderate the mediating effect of “R - TSMU→TKS→TC”; thus confirming **H6b**.

**TABLE 5:** Results of moderated mediating effect test.

Effects	Estimate	*SE*	95% confidence interval		
			Lower limit		Upper limit
**W-TSMU→TKS→TC**					
Low TMX (–1 SD)	0.086	0.098	–0.066		0.333
High TMX (+1 SD)	0.629	0.179	0.335		1.035
Differences between the two groups	0.542	0.198	0.224		1.010
**R-TSMU→TKS→TC**					
Low TMX (–1 SD)	–0.065	0.178		–0.425	0.279
High TMX (+1 SD)	1.094	0.202		0.703	1.491
Differences between the two groups	1.159	0.282		0.602	1.748

*The difference between the two groups is equal to the mediating effect of conditions under high TMX minus the mediating effect of conditions under low TMX. W-TSMU, work-related TSMU; R-TSMU, relationship-related TSMU; TKS, team knowledge sharing; TC, team creativity.*

## Discussion

The past few years have witnessed growing academic interest in social media usage (e.g., Brooks, 2015; Haddud et al., 2016; Van Zoonen et al., 2017; Sun et al., 2019; Liu et al., 2020; Ma et al., 2020), but few studies have focused on the relationship between TSMU and team creativity (e.g., Leonardi, 2014; Cheng and Krumwiede, 2018; Wushe and Shenje, 2019; Chen et al., 2020; Korzynski et al., 2020). Basing on communication visibility theory and social exchange theory, this study craft a theoretical framework to understand how and when TSMU exerted positive effect on team creativity. The results of empirical study support the proposed research model, and the main findings are as follows:

First, we found that both work-related TSMU and relationship-related TSMU are positively related to team creativity. According to communication visibility theory, TSMU could facilitate team creativity by message transparency and network translucence (Leonardi, 2014). Work-related TSMU could increase message transparency which facilitates team members to generate ideas, coordinate with each other, and complete tasks, whereas relationship-related TSMU could enhance network translucence which allows team members to build and maintain network relationships, so as to enhance team cohesion that positively related to team creativity (Joo et al., 2012).

Second, we found that team knowledge sharing partially mediates the relationship between work-related TSMU and team creativity. Based on communication visibility theory, social media usage in work teams makes team members accurately recognize their advantages of professional knowledge and information resource, and then share knowledge and information in the team, which enables the team to integrate the resources to accomplish team tasks creatively, such as developing new products or procedures. The present findings also show that team knowledge sharing partially mediates the relationship between relationship-related TSMU and team creativity. Relationship-oriented TSMU refers that team members use social media to encourage or support colleagues, so as to promote the sharing of knowledge within the team, thus leading to enhanced team creativity.

Third, the indirect effect of TSMU on team creativity via team knowledge sharing was moderated by TMX. Based on social exchange theory, Low levels of TMX may reflect troubled relationships with other team members, and high levels of TMX may reflect more congenial and more reliable coworker relationships. In the situation of work-related TSMU, team members with high-quality TMX are more willing to share knowledge to promote the completion of team tasks. Meanwhile, in the situation of relationship-related TSMU, team members with high-quality TMX are more willing to actively pay attention to colleagues through social media, be aware of their interests and hobbies, and share knowledge with them more actively. That is, the higher the TMX, the greater the mediating effect of team knowledge sharing between work-related TSMU and team creativity. While the higher the TMX, the greater the mediating effect of team knowledge sharing between relationship-related TSMU and team creativity.

### Theoretical Implications

First, this study contributes to social media usage and team creativity literature by revealing the relationship between TSMU and team creativity. Prior research has shown a relationship between social media usage and individual creativity (Cheng and Krumwiede, 2018; Bhimani et al., 2019; Chen et al., 2020; Korzynski et al., 2020). However, few literature focused on the relationship of TSMU and team creativity. This study focused on work teams, and defined TSMU which are divided into two types: work-related TSMU and relationship-related TSMU. The motivation of work-related TSMU focuses on completing team work efficiently, whereas the motivation of relationship-related TSMU emphasizes building and maintaining team members’ relationships. Furthermore, this study examined the relationship of work-related TSMU and team creativity, and the relationship of relationship-related TSMU and team creativity. The empirical results show that, both work-related TSMU and relationship-related TSMU are positively related to team creativity. The findings enrich the literature on social media usage and team creativity.

Second, this study enriches the application of communication visibility theory, and extends the current understanding of the mediating mechanism of TSMU on team creativity. Leonardi (2014) had proposed communication visibility theory, which provides a new perspective for the research fields of organizational behavior and knowledge management (Flyverbom et al., 2016). However, few researchers conducted empirical tests on it (Engelbrecht et al., 2019). Considering team creativity is the aggregation of ideas generated by individual members, the effect of TSMU on team creativity is a more complex process. Basing on communication visibility theory, this study explores the mediating effect of team knowledge sharing between TSMU and team creativity. Communication visibility enabled by TSMU leads to enhanced awareness of “who knows what” and “who knows whom” within the team, which not only enables team members to exchange work-related knowledge and experience, but also facilitates them to encourage and support with each other. In this way, the knowledge stock of the team is increasing, and sufficient knowledge resources help to improve the team creativity. The findings indicate that team knowledge sharing partially mediates the relationship between work-related TSMU and team creativity and that between relationship-related TSMU and team creativity. These findings contribute to communication visibility theory by revealing the mediating role of team knowledge sharing between TSMU and team creativity.

Third, this study extends the research of the boundary conditions under which the mediating effect of team knowledge sharing between TSMU and team creativity are strong or weak. Based on social exchange theory, TMX reflects the quality of relationships within a team. Since previous studies have confirmed that TMX is related to knowledge sharing, high-quality TMX relationship helps to create an atmosphere of mutual assistance and reciprocity, so as to effectively stimulate individuals’ willingness to share knowledge (Baek et al., 2018; Chen, 2018). In addition, this study was conducted in China. In the context of Chinese culture, the perceived relationship quality of individuals will have an important impact on the motivation of individual behavior. Therefore, this study introduces TMX as an moderator to explore how TMX affects the relationship between TSMU and knowledge sharing. The findings indicate that TMX positively moderates the indirect effect of work-related TSMU on team creativity via team knowledge sharing, and TMX positively moderates the indirect effect of relationship-related TSMU on team creativity via team knowledge sharing. These findings not only expands the boundary conditions of TSMU on team creativity via knowledge sharing, but also extends the literature on the relationship of TSMU, TMX and team knowledge sharing in the Chinese context.

### Practical Implications

First, the findings show that TSMU has not only a direct effect on team creativity but also an indirect effect on team creativity via TMX. Thus, for team leaders, it is wise to encourage team members to use social media for both work-related and relationship-related purposes. On the one hand, to prompt the usage of social media for work-related purposes, the work team should use social media platforms such as QQ and Wechat to establish an online work group. Relying on the online working group, the team leader can post information such as task objectives, task allocation scheme, and task progress arrangement to the work group so that team members can understand who knows what within the team, which further facilitates team members to exchange knowledge and ideas. This then leads to integration of existing knowledge and ideas within teams to enhance team creativity. On the other hand, relationship-oriented TSMU makes team members more willing to actively express their ideas or opinions to teammates. Thus, the team leader should encourage team members to use social media for encouraging teammates when they are in trouble or communicating interests and hobbies so as to enhance trust among team members and lead to more exchanging of knowledge sharing within teams.

Second, given the crucial mediating role of team knowledge sharing on the relationship between TSMU and team creativity, knowledge sharing can be an effective knowledge management tool influencing team creativity (Bodla et al., 2016; Ratasuk and Charoensukmongkol, 2020). Organizations should take multiple ways to encourage team knowledge sharing. Knowledge sharing should be systematically embedded into organizations’ employee well-being program so as to encourage, recognize, and reward employees who share knowledge to prompt collaboration in work task.

Third, given the crucial moderating role of TMX on the relationship of TSMU on team creativity through team knowledge sharing, team leaders should pay attention to improving the quality of TMX. Organizations can take multiple ways to improve TMX among team members. One approach is for team leaders to organize regular team-building activities (e.g., retreats, reading clubs, and sport activities) to create opportunities for teammates’ interactions (Rutishauser and Sender, 2019). In addition, organizations should establish work spaces where teams spend time together so as to strengthen friendship within teams. Moreover, organizations should consider organizing training opportunities for team members to develop the necessary competencies to engage in high-quality TMX relationships.

### Limitations and Future Research

Despite its contribution to theory and practice, this study still has several potential limitations. First, basing on communication visibility theory, this study confirms that TSMU not only directly but also indirectly and positively affect team creativity. However, social media usage overload occurs when workers are interrupted by too many communication requirements through various media, such as email, instant message, and so on. Some studies have provided empirical evidence that social media usage overload is positively related to social network fatigue (Lee et al., 2016; Zhang et al., 2016). Therefore, TSMU may be a double-edged sword, and both the positive and negative effect mechanisms of TSMU should be studied. Future research can explore the curvilinear relationship between TSMU and team creativity.

Second, TSMU not only makes the communication within the team more convenient but also makes it easy for the team to obtain external resources. Both internal knowledge and external resources are necessary for innovation, but this study only discusses the mediating role of team knowledge sharing between TSMU and team creativity. Future research should take steps to investigate the dual mediation mechanism of external knowledge search and internal knowledge integration on the relationship between TSMU and team creativity.

Third, this study explores the moderating role of TMX between TSMU and team knowledge sharing, but ignores TSMU may be a potential antecedent of TMX. In order to better examine the moderating effect of TMX, future research should measure TMX at Time 1, and TSMU measured at Time 2 in the questionnaire survey. In other words, if the study measure TMX before TSMU, thus the moderating effect of TMX will be more meaningful.

## Data Availability Statement

The raw data supporting the conclusions of this article will be made available by the authors, without undue reservation.

## Ethics Statement

As a protection of all participants, all subjects read informed consent before participating in this study and voluntarily made their decision to complete surveys. The protocol was approved by an institutional review board in Xiangtan University of China.

## Author Contributions

HW developed the theoretical model and wrote the manuscript. YX was responsible for empirical analysis. XS collected the data, analyzed the data, and participated in manuscript writing. XL participated in manuscript writing. All authors contributed to the article and approved the submitted version.

## Conflict of Interest

The authors declare that the research was conducted in the absence of any commercial or financial relationships that could be construed as a potential conflict of interest.

## Publisher’s Note

All claims expressed in this article are solely those of the authors and do not necessarily represent those of their affiliated organizations, or those of the publisher, the editors and the reviewers. Any product that may be evaluated in this article, or claim that may be made by its manufacturer, is not guaranteed or endorsed by the publisher.
